# Effectiveness of a Flexible and Continuous Case Management Program for Suicide Attempters

**DOI:** 10.3390/ijerph17072599

**Published:** 2020-04-10

**Authors:** Min-Hyuk Kim, Jinhee Lee, Hyunjean Noh, Jin-Pyo Hong, Hyun Kim, Yong Sung Cha, Joung-Sook Ahn, Sei-Jin Chang, Seongho Min

**Affiliations:** 1 Department of Psychiatry, Yonsei University Wonju College of Medicine, Wonju 26426, Korea; mhkim09@yonsei.ac.kr (M.-H.K.); jinh.lee95@yonsei.ac.kr (J.L.); jsahn@yonsei.ac.kr (J.-S.A.); 2 Hopefull Psychiatric Clinic, Anyang, Korea; no10011@hanmail.net; 3 Department of Psychiatry, Samsung Medical Center, Sungkyunkwan University School of Medicine, Seoul 06351, Korea; suhurhong@gmail.com; 4 Department of Emergency Medicine, Yonsei University Wonju College of Medicine, Wonju 26426, Korea; khyun@yonsei.ac.kr (H.K.); emyscha@yonsei.ac.kr (Y.S.C.); 5 Department of Preventive Medicine, Yonsei University Wonju College of Medicine, Wonju 26426, Korea; chang0343@yonsei.ac.kr

**Keywords:** suicide, suicide attempts, intervention, case management

## Abstract

The purpose of this study was to investigate the effect of continuous case management with a flexible approach on the prevention of suicide by suicide reattempt in a real clinical setting. The subjects in this study were 526 suicide attempters who visited emergency rooms in a teaching hospital in South Korea. Subjects were provided a continuous case management program with a flexible approach according to the severity of their suicide risk and needs. During the entire observation period (from 182 days to 855 days, mean = 572 ± 254), 18 patients (3.7%) died by suicide reattempt: Eight patients (2.27%) in the case management group and 10 patients (7.35%) in the no-case management group. The Cox regression analysis showed that the case management group had a 75% lower risk of death from suicide attempts than the no-case management group (HR = 0.34, 95% CI = 0.13–0.87). This result was shown to be more robust after adjusting for confounding factors such as gender, age, psychiatric treatment, suicide attempts, and family history of suicide (adjusted HR = 0.27, 95% CI = 0.09–0.83). This study was conducted in a single teaching hospital and not a randomized controlled one. A flexible and continuous case management program for suicide attempters is effective for preventing death by suicide reattempts.

## 1. Introduction

Suicide is a complex major public health issue and accounts for an estimated 11.4 suicides per 100,000 people that occur per year, resulting in 804,000 deaths worldwide every year [1]. The suicide rate in Korea is the highest among the Organization for Economic Co-Operation and Development (OECD) member countries with 29.1 deaths per 100,000 people, which is approximately 2.3 times higher than the OECD average of 12.5 deaths per 100,000 people [2]. Strategies to prevent suicide include common prevention for the general population (education on suicide, publicity, access control in lethal methods of suicide), selective prevention for people vulnerable to suicide (people with mental illnesses, etc.), and intensive prevention for high-risk groups such as people with mental illnesses known to be associated with suicide or those who have already attempted suicide [3,4]. In particular, suicide attempters even once have 66 times higher likelihood of dying by suicide within a year of the first attempt, 16% (12%–25%) in a year, 21% (12%–30%) in 1–4 years, and 23% (11%–32%) after four years and above make a reattempt [5,6]. According to a study that followed up suicide attempters for 37 years, 13% of suicide attempters died by suicide reattempts, and deaths caused by suicide reattempts were observed even 20~30 years after suicide attempts [7].

Previous suicide attempts are the most potent predictors of suicide and suicide attempts [8,9,10]. Therefore, management following suicide attempts is an important strategy for suicide prevention. Several studies have examined various methods in this regard, such as telephone contact, postcards, and case management [11,12,13,14,15,16,17,18,19,20,21,22]. However, the results are mixed. Some studies reported that interventions such as sending postcards and a telephone contact have effects on reducing the suicide rate or reattempt rate [5,6,8,12], while other studies do not [4,20]. Case management has been shown to be the most comprehensive method for taking care of suicide attempters, yet previous research results in this respect have remained inconclusive [14,16,17,18,21].

A recent meta-analysis has shown that case management is not effective in reducing suicide attempts or suicide, but there are some considerations in interpreting these results [23]. The relatively small number of studies, small sample size, and various settings that the studies were conducted make it hard to draw a firm conclusion. Although a randomized controlled trial (RCT) may be the best method for evaluating the effectiveness of a treatment, it may not reflect the actual situation owing to ethical and methodological problems in suicide attempt research. The “treatment as usual” or “enhanced care” used as a control in RCTs is likely to be more intensive than actual care, possibly reducing the difference owing to the effect of case management [18]. There is also the possibility that the fixed intervention interval or duration used in the RCT will affect the outcome. In a large-scale study, the Action J study, case management was effective in reducing suicide reattempts up to six months, and the effect has since vanished. It was estimated that the reason for the lack of effectiveness was the increase in the interval of case management [17]. In addition, most RCTs are likely to fail to appropriately and adequately reflect the various changes and needs of suicide attempters in real life due to their strict protocols [16]. This is considered to be a limitation of this research due to the unique situation of suicide. This possibility can be speculated when considering the fact that studies conducted in real clinical settings of a community show positive results [16,21,22]. In addition, previous studies have mainly focused on suicide reattempts as the primary outcome, although the ultimate primary outcome of suicide prevention is suicide [14,21].

We hypothesized that the continuous case management program for suicide attempters who visited the emergency room reduces suicide mortality by reattempts. The purpose of this study was to investigate the effect of continuous case management with a flexible approach on suicide reduction in a real clinical setting.

## 2. Material and Methods

### 2.1. Methods

#### 2.1.1. Subjects

**Figure 1:**
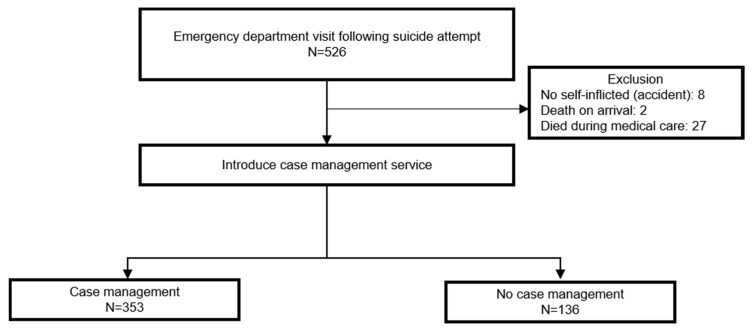
Flow chart of the study participants.

The study subjects were 526 suicide attempters who visited emergency rooms in a teaching hospital in South Korea between 10 March, 2009 and 31 December, 2011. A total of 489 suicide attempters were finally analyzed, excluding those who had died on arrival (*n* = 2), those who died during treatment due to a suicide attempt (*n* = 27), and those who were considered suicide attempters initially but turned out to be not suicide attempters later after a thorough interview (*n* = 8) (Figure 1).

#### 2.1.2. Procedures and Intervention

The suicide attempters were accessed by psychiatrists as soon as possible after being established as medically stable. After their psychosocial assessment, the first contact was made as soon as possible by a face-to-face interview with case managers. All suicide attempters were introduced to the program by case managers. Suicide attempters were assigned to the case management group if they consented and to the no-case management group (treatment as usual) if they did not consent.

Case managers comprised trained nurses or social workers. The procedures and contents of case management were guided and supervised once a week by two board certified psychiatrists (M.-H.K. and S.M.). The guidance and supervision of case management and the determination and solutions of problems were carried out in case management meetings every week.

**Figure 2:**
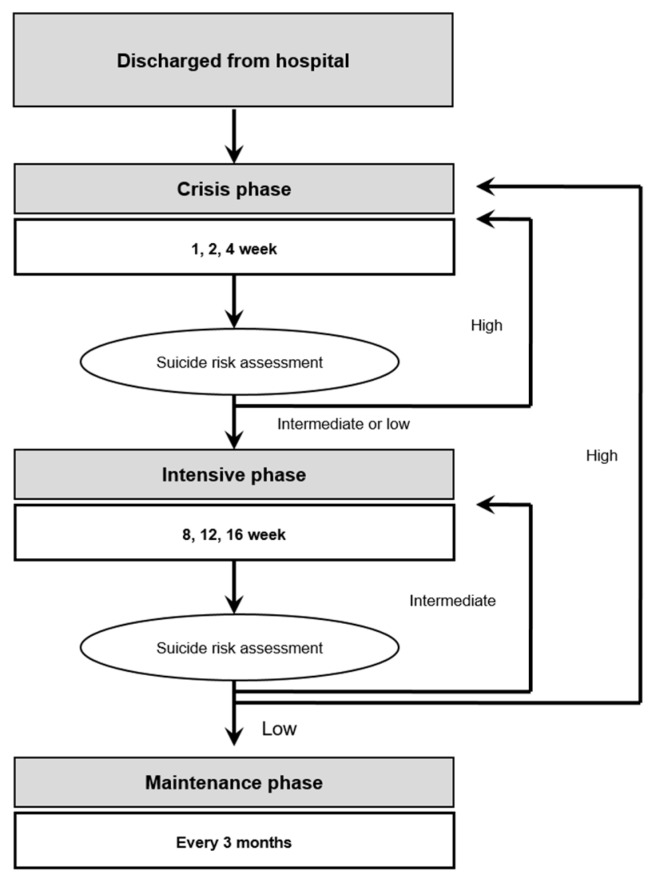
Protocol of the case management program.

The case management was divided into three phases: The crisis, intensive, and maintenance phase (Figure 2). According to the protocol, the first follow-up contact with suicide attempters was conducted within at least three days of their visit to the emergency room, where they underwent crisis management until week 4. Every contact was conducted in person or via a phone according to the situation and convenience of subjects.

- Crisis management and suicide risk were conducted in weeks 1, 2, and 4. If the suicide risk was high, the attempters underwent crisis management again, but if the risk was low, the attempters went through intensive management until week 6.
- Intensive management constituted case management conducted in weeks 8, 12, and 16, and suicide risk was assessed along with intensive management. If the suicide risk was high, the attempters went back to crisis management; if the risk was moderate, they went back to intensive management; and if the risk was low, they went to the maintenance phase, in which case management was conducted every three months.
- Maintenance was conducted continuously unless the patients refused. The shift between case management phases was carried out after the risk through the most recently conducted systematic suicide risk assessment was verified. If the patients seemed stable after one year and eight months, the frequency of contact was reduced to once every six months, and mail correspondence was sent continuously.

The contents of the case management program are as follows. First, there is a psychotherapeutic element, such as briefly saying hello and showing interest in the suicide attempters, asking them to talk about their difficulties, empathizing with them, and supporting them emotionally. Second, there is an evaluative element in which the risk of suicide, psychiatric symptoms such as depression, and the client’s general condition are evaluated. Third, there is an educational element, which includes explaining the necessity of and recommending psychiatric treatment, assessing compliance, promoting the motivation for therapy, and monitoring treatment. Fourth, there is a problem-solving element to solve the issues faced by the patient. Fifth, there is an information-providing element of offering guidance on medical care centers, local medical institutions, and connection to relevant agencies. Sixth, there is an element regarding suicide attempters’ family members, who commonly feel guilt and a psychological burden. Such negative emotions felt by family members induce suicide attempters to deny, avoid, and diminish the problem, which may be a factor that hinders therapy for suicide attempters. Therefore, case management can provide emotional support for families. Seventh, a support system is determined within the family, and resources are developed to help suicide attempters since their families commonly have no support system or a poor one. Eighth, case management educates families in obtaining skills and methods to help suicide attempters, since there are cases in which families cannot properly help them owing to a dysfunctional communication structure or ambivalent emotions toward the patient, rather stimulating emotions to trigger suicide. Ninth, by offering education about prior warning signals of suicide attempts and the risks of suicide, families can perceive suicide reattempts in advance and help patients receive treatment at an early stage if a risk is detected. Tenth, case management provides education about the necessity of psychiatric treatment through the family and increases the compliance of patients who need therapy through monitoring.

In the no-case management group, patients were provided treatment of usual care that was identical with the treatment in the case management group except case management program. The treatment of usual care included psychosocial assessment, psychiatric interview, and education at an emergency room plus arranging the schedule for out-patient psychiatric clinic.

All procedures contributing to this work complied with the ethical standards of the relevant national and institutional committees on human experimentation and with the Helsinki Declaration of 1975, as revised in 2008. This study was approved by the Institutional Review Board (YWMR-14-0-008).

### 2.2. Measures

#### 2.2.1. Socio-Demographic Characteristics and Suicide-Related Factors

Basic socio-demographic data were collected from the participants who could be interviewed among the suicide attempters that visited the emergency rooms. A history of mental disease, comorbid diseases, medication, familial psychiatric illness, and smoking and drinking status were also investigated. Moreover, recent psychosocial stress was also identified to determine patients’ mental symptoms, such as the patient’s depression and insomnia or the direct or indirect psychological cause of suicide attempts.

Regarding suicide-related factors, suicide plan, time, suicidal thoughts, suicidal motivation, suicide attempts in the past, and family history of suicide were investigated.

#### 2.2.2. Systematic Suicide Risk Assessment

The systematic suicide risk assessment is a kind of checklist for risk factors and protective factors that a patient may have. The raters make the decision on the degree of suicide risk for a patient based on the checklist and clinical judgement. The systematic suicide risk assessment was categorized into five factors: Individual, clinical, interpersonal, situational, and demographic. The interviewer assessed each of the risk and defensive/protective factors and classified them into high, medium, and low influence, while assessing and recording the total risk score into high, medium, and low [24].

#### 2.2.3. Identification of Death by Suicide

For the data of deaths from suicide reattempts, this study used the data of X60–X84, which is the code number for suicides based on the data from Statistics Korea from March 2009 to December 31, 2011. The data were provided by linkage to the database of the National Statistics Office. For those who were dead, causes of death were also established through linkage to the database of the National Statistics Office. This data and hospital records were matched using the unique national identification number assigned to all Korean citizens. All deaths in Korea are reported to the National Statistics Office by a document of death notice, which contains the cause of death [25].

#### 2.2.4. Statistical Analysis

Characteristics of case management consenters and nonconsenters were divided into socio-demographic, clinical, and suicide-related variables, which were compared through a chi-square analysis or Fisher’s exact test for dichotomous variables and a t-test for continuous variables. The multiple logistic regression analysis was conducted to analyze the characteristics of the case management consenters.

A survival analysis was conducted using the Kaplan–Meier method to compare the point of death in the suicide reattempts of the consenters and nonconsenters. Furthermore, the Cox proportional hazards model was used to analyze the variables with a significant impact on lethality in suicide reattempts in order to determine the impact of case management on lethality. Survival data were used for processing if the participants were continuously receiving case management (*n* = 129), whereas censored data were used for the loss of data in a follow-up survey, such as connection to other institutes (*n* = 3) and death by chronic illness (*n* = 11).

All statistical analyses were conducted using SPSS (version 20.0, IBM Corp., Armonk, NY, USA), and the results were considered statistically significant only at *p* < 0.05.

## 3. Results

### 3.1. Characteristics of Participants in the Case Management Program

Seventy-two percent (353/489) of all suicide attempters participated in the case management program. By the end of the observation period (31 December, 2012), 199 people (56.7%) among the cases remained in the case management program (24 people were referred to another mental health center because they moved to another city, 113 people refused to receive the program). The mean duration of case management was 397 days (SD 262), and the median was 364 days. There were no statistically significant differences in age, gender, education, marital status, and religion between the case management group and the no-case management group (Table 1).

The case management group had a greater prevalence of family history of suicide, past history of psychiatric treatment, and ongoing psychiatric treatment than the no-case management group. The case management group also used less cutting than the no-case management group (*p* = 0.008) (Table 2).

### 3.2. Effectiveness of the Case Management Program on Death by Suicide Reattempt

During the entire observation period (from 182 days to 855 days, mean = 572 ± 254), 18 patients (3.7%) died by suicide reattempt (eight patients (2.27%) in the case management group, and 10 patients did so (7.35%) in the no-case management group). Fifty percent of the deaths occurred within six months in the case management group and within a month in the no-case management group.

When comparing subjects who died by suicide reattempt with who did not die by suicide reattempt, elderly and males were more frequent in the group of subjects who died by suicide reattempt.

**Figure 3:**
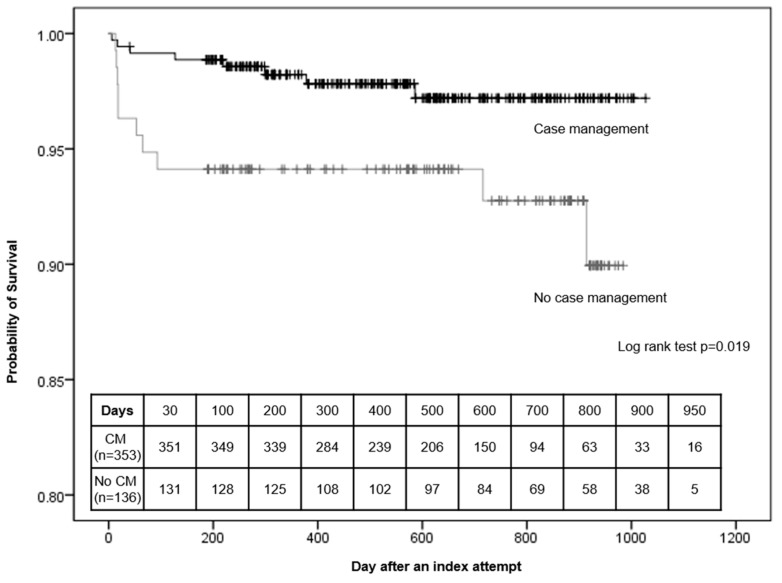
Survival curves by case management program. CM: Case management.

A Kaplan–Meyer plot is shown in Figure 3, and the log-rank test revealed a significant difference in favor of fewer deaths by suicide in the case management group (*p* = 0.019). The Cox regression results showed that the case management group had a 75% lower risk of death from suicide attempts than the no-case management group (HR = 0.34, 95% CI = 0.13–0.87). This result was more robust after adjusting for confounding factors such as gender, age, psychiatric treatment, suicide attempts, and family history of suicide (adjusted HR = 0.27, 95% CI = 0.09–0.83) (Table 3).

## 4. Discussion

In this study, we investigated the effectiveness of case management for suicide attempters visiting an emergency room in a real clinical situation.

Our results indicated that the case management program reduced the risk of death from suicide reattempts by 75% and delayed the time to death from suicide attempts. In addition, the results remained the same even after controlling for other confounding variables and were even more robust. Pan et al. showed that a nationwide aftercare program was effective in reducing mortality from suicide attempts and that the risk was reduced by 63.5% in people with initial willingness to use the service, which is comparable to the results of our study [21].

### 4.1. Effectiveness of a Flexible, Continuous Case Management Program

In a real clinical situation, there are several reports that an aftercare program is effective, as our results show [21,22], but RCT studies show no differences, apart from the study by Hvid et al. [16,17,18]. There are some possible explanations for this discrepancy. First, in a realistic clinical situation, a case management program is more flexible depending on patients’ needs. Interestingly, only one RCT study with a positive result has emphasized a flexible approach [16]. Second, in an RCT study, it may be possible that the treatment as usual or the standard treatment has provided itself with a mild level of case management, especially with a continuous follow-up of the control group [18]. In addition, subjects of an RCT may be somewhat different from the target group in a real clinical setting, as it may provide a case management service to all suicide attempters regardless of the presence of mental illness in a real-world situation. Third, the frequency and duration of case management service may influence the results. Continuous case management may have contributed to the sustained effect. The results of some studies have shown that the initial effectiveness of intervention is not necessarily retained as the frequency of intervention decreases, and they thus suggest the need for continuous management [17,19]. The risk of suicide reattempts is the highest in the first year [5,26], but there is a continuing risk afterward, so it is reasonable to expect that ongoing case management will be effective in preventing suicide. Then, how long should the case management be maintained for suicide prevention? The long-term case management might increase case-loading per case-managers with limited resources in the community, in turn, weaken the effect of the case management program. Reducing the risk of suicide with limited resources is important and a difficult aspect of the continuous case management program. We could not determine and provide an optimal duration of the case management program from the result of this study.

We propose the basic components for an effective case management program for suicide attempters based on the results of previous studies and ours.

1. Case management programs can be flexible depending on the patient’s needs [16].
2. The frequency and duration of case management programs need to be modifiable according to the level of suicide risk while balancing the limitations of available resources [17,19].
3. The first contact should be made as soon as possible by face-to-face interviews in person [12,16,21].

Further RCT studies including these components are needed to confirm the effectiveness of case management programs for suicide prevention.

### 4.2. Death by Suicide

We should note that the mortality rate by suicide reattempts in the 572-day observation period was 3.7% for the whole sample and 7.35% for the no-case management group. The mortality rate by suicide reattempts in the present study is higher than that of the 2013 national survey in South Korea, where 2.7% died from complete suicide during the average three-year observation period [26]. It is also higher than the suicide mortality rate in Western countries and in Taiwan, and it is comparable to that in Japan [10,17,21]. The high mortality rate by suicide reattempts in this study is presumed to reflect the characteristics of the region where research hospital was located with a high mortality rate from suicide, 45.2 per 100,000 in 2011 [2]. However, it is uncertain whether case management programs constitute a more effective intervention in a region with a high suicide mortality rate.

In addition, death by suicide reattempts in the no-case management group was observed even two years after the index attempt, which is a reason why continuous case management is important. This is consistent with a previous study suggesting that intervention for male suicide attempters should be extended up to at least 20 months [26].

### 4.3. Limitations and Strengths

This study has the benefit from a relatively large sample based on data collected from patients visiting the ED.

However, several limitations apply to the present study. First, this study was conducted in a single teaching hospital, although the study site covered the entire regional area. Therefore, generalization to other populations or regions is limited. Our study was not an RCT. Although we tried to control for certain confounding variables that were identified, it is possible that other variables that we could not have controlled for influenced our result. Second, sensitivity analyses or subgroup analyses to examine factors related to the prevention of suicide could not be conducted because of the small sample size. Finally, the observation period was relatively short. Research is needed to determine the long-term outcome. The use of National Statistics Office data on suicide as the main outcome is one of the strengths of this study since the suicide rate is the most important and fundamental outcome of interventions for suicide prevention.

## 5. Conclusions

In conclusion, this study showed that the long-term case management program for suicide attempters who visited the emergency room reduced the risk of suicide mortality by reattempts. The result suggests that a case management program could be one of the effective strategies for suicide prevention of suicide attempters. In addition, we propose that flexible and continuous management with early contact after discharge could be the basic component of a case management program for suicide attempters.

## Figures and Tables

**Table 1:** Demographic characteristics of the participants.

Variables	Case Management	No-Case Management	χ2	*p*-Value
	(*n* = 353)	(*n* = 136)		
Age			1.576	0.665
>24	52 (14.7%)	16 (11.8%)		
25–44	127 (36.0%)	56 (41.2%)		
45–59	93 (26.3%)	36 (26.5%)		
60+	81 (22.9%)	28 (20.6%)		
Gender			0.200	0.655
Male	135 (38.2%)	55 (40.4)		
Female	218 (61.8%)	81 (29.6)		
Education (20) ^a^			1.573	0.455
Elementary school	106 (30.5%)	32 (26.2%)		
Middle/High school	200 (57.6%)	71 (58.2%)		
College	41 (11.8%)	19 (15.9%)		
Marital status (4) ^a^			4.110	0.128
Single or never married	84 (23.8%)	31 (23.5%)		
Married/Cohabitation	204 (57.8%)	66 (50.0%)		
Separated/Divorced/Widowed	65 (18.4%)	35 (26.5%)		
Religion (21) ^a^			0.245	0.620
No	179 (52.2%)	62 (49.6%)		
Yes	164 (47.8%)	63 (50.4%)		
Somatic illness (186) ^a^			0.276	0.600
No	158 (65.3%)	42 (68.9%)		
Yes	84 (34.7%)	19 (31.1%)		

^a^ Numbers in parenthesis mean missing data (missing data occurred by no response to the questions by the patients).

**Table 2:** Clinical and suicide-related characteristics of the participants.

Variables	Case Management	No-Case Management	χ2	*p*-Value
	(*n* = 353)	(*n* = 136)		
Previous suicide attempts (5) ^a^			1.395	0.237
No	259 (73.4%)	89 (67.9%)		
Yes	94 (26.6%)	42 (32.1%)		
Family history of suicide (17) ^a^			6.592	0.010
No	316 (90.3%)	119 (97.5%)		
Yes	34 (9.7%)	3 (2.5%)		
Family history of psychiatric disorders (21) ^a^			1.514	0.219
No	309 (89.8%)	116 (93.5%)		
Yes	35 (10.2%)	8 (6.5%)		
History of psychiatric treatment (22) ^a^			11.117	0.001
No	194 (56.9%)	93 (73.8%)		
Yes	147 (43.1%)	33 (26.2%)		
Ongoing psychiatric treatment (22) ^a^			5.335	0.021
No	249 (73%)	105 (83.3%)		
Yes	92 (27.0%)	21 (16.7%)		
Use of alcohol before the index attempts (6) ^a^			3.765	0.052
No	174 (49.7%)	53 (39.8%)		
Yes	176 (50.3%)	80 (60.2%)		
Psychiatric evaluation at ER			2.603	0.107
No	108 (30.6%)	52 (38.2%)		
Yes	245 (69.4%)	84 (61.8%)		
Current suicidal idea (133) ^a^			0.958	0.328
No	142 (54.6%)	58 (60.4%)		
Yes	118 (45.4%)	38 (39.6%)		
Planned suicide attempts (198) ^a^			1.296	0.255
No	192 (82.4%)	44 (75.9%)		
Yes	41 (17.6%)	14 (24.1%)		
Psychiatric diagnosis (31) ^a^			7.696	0.103
Mood disorder	157 (45.9%)	45 (38.8%)		
Psychotic disorder	10 (2.9%)	1 (0.9%)		
Substance use disorder	53 (15.5%)	19 (16.4%)		
Others	99 (28.9%)	35 (30.2%)		
No axis I disorder	23 ((6.7%)	16 (13.8%)		
Motivation for suicide attempt				
Interpersonal problem	236 (66.9%)	82 (60.3%)	1.859	0.204
Economic or job problem	46 (13.0%)	17 (12.5%)	0.025	0.875
Separation problem ^b^	8 (2.3%)	3 (2.2%)		1.000
Mistreatment or violence problem	62 (17.6%)	28 (20.6%)	0.598	0.439
Psychiatric problem ^b^	8 (2.3%)	5 (3.7%)		0.346
Method of attempt (2) ^a^			19.122	0.008
Poisoning	293 (83.0%)	102 (76.1%)		
Cutting	11 (3.1%)	15 (11.2%)		
Stabbing	5 (1.4%)	3 (2.2%)		
Drowning	1 (0.3%)	1 (0.7%)		
Hanging	19 (5.4%)	3 (2.2%)		
Asphyxia	16 (4.5%)	4 (3.0%)		
Falling from a height	3 (0.8%)	4 (3.0%)		
Others	5 (1.4%)	2 (1.5%)		
Psychiatric Treatment within three months following index attempt			1.349	0.245
No	271 (76.6%)	111 (81.6%)		
Yes	82 (23.2%)	25 (18.4%)		
Duration of case management		-		
Median (IQR)	364 (210–587)	-		
Mean (SD)	397.99 (262)	-		

^a^ Numbers in parenthesis mean missing data (missing data occurred by no response to the questions by the patients); ^b^ conducted by Fisher’s exact test.

**Table 3:** Results of Cox regression analysis for death by suicide.

	HR	95% CI	aHR1	95% CI	aHR2	95% CI
Case management	0.341	0.133–0.872	0.306	0.118–0.790	0.266	0.085–0.834

aHR1: Adjusted for age and sex; aHR2: Adjusted for age, sex, ongoing psychiatric treatment, methods of suicide attempt, and family history of suicide attempt; reference group, no case management.
